# Developmental Mutators and Early Onset Cancer

**DOI:** 10.3389/fped.2020.00189

**Published:** 2020-04-28

**Authors:** Alex Kentsis, Steven A. Frank

**Affiliations:** ^1^Sloan Kettering Institute and Department of Pediatrics, Weill Medical College of Cornell University and Memorial Sloan Kettering Cancer Center, New York, NY, United States; ^2^Department of Ecology and Evolutionary Biology, University of California, Irvine, Irvine, CA, United States

**Keywords:** cancer genetics and genomics, developmental plasticity, mosaicism, childhood cancer, prevention

## Abstract

A new hypothesis suggests that somatic genome remodeling during normal development can cause mutations that explain many early onset cancers in children and adults.

Currently, only about 10% of early onset cancers can be explained by known inherited cancer predisposition, environmental mutagens or oncogenic pathogens (1–4). What explains the majority of cancers of children and young adults? We suggest that developmental mutators may be important. A developmental mutator is a genomic process active during normal tissue development that promotes specific somatic mutations. Enhanced mutations during development lead to tissue-specific somatic mosaicism, because mutations during development propagate to all descendant cells. A mosaic individual who carries a significant fraction of mutated cells has an elevated risk of early onset cancer (5), especially if the mutation process tends to affect proto-oncogenes or tumor suppressor genes that regulate developmental processes.

For example, the RAG1/2 DNA recombinase mediates somatic DNA rearrangements of immunoglobulin receptor genes during normal B- and T-cell development (6). This genomic rearrangement process also promotes “off-target” deletions and other aberrant chromosomal rearrangements. Leukemias and lymphomas have specific somatic deletions and chromosomal translocations that exhibit signatures of RAG1/2 activity and related mutational processes (7). Indeed, some of these somatic mutations are detected in the blood of healthy individuals, consistent with their somatic mosaic induction during development.

We suggest that a substantial fraction of early onset cancers arise from tissue-specific developmental genome remodeling and somatic mosaicism induced by specific developmental mutators acting during childhood and young adulthood. In addition to leukemias and lymphomas, this applies to sarcomas, medulloblastomas, neuroblastomas, and various other cancers that affect children and young adults.

Recent genomic surveys of human cancers have revealed a diverse set of oncogenic mutations (8). In particular, tumors in older individuals exhibit relatively high rates of genome-wide nucleotide substitutions and overall mutation rates. By contrast, the majority of tumors with early onset have nucleotide substitution rates that are on average indistinguishable from their corresponding normal tissues. Instead of high overall mutation rates, these early onset tumors tend to exhibit regional mutations with distinct sequence features that dysregulate specific oncogenes and tumor suppressor genes.

For example, the majority of chromosomal translocations, deletions and amplifications in medulloblastomas, neuroblastomas, ependymomas, Ewing, and various other sarcomas that affect children and young adults tend to be associated with chromothripsis, chromoplexy, or other mutational processes with distinct structural features (9). Likewise, osteosarcomas, retinoblastomas, and Wilms tumors exhibit distinctive chromosomal rearrangements and mutations, involving both unique genes and general tumor suppressor and oncogenes.

The distinctive mutational spectra of early onset cancers suggest that specific processes may drive their progression. Our developmental mutator hypothesis provides a potential explanation. In particular, specific cancers in childhood or young adulthood may often arise from the activation of endogenous DNA nucleases or biochemical processes that induce mutations or abrogate normal DNA repair systems during specific developmental periods. While all tumors result from complex evolution involving diverse mutational processes that can be shared, specific mutational processes are predicted to be responsible for the early-onset induction of distinct tumors in children and young adults.

Developmental mutators cause additional mutations beyond those that normally arise from aging and environmental exposure. Those excess mutations affecting children and young adults suggest an evolutionary cost of developmental mutators that is likely balanced by the benefits of genomic modifications during the early stages of cell lineage differentiation. This distinguishes them from other mutational processes that occur upon aging and environmental exposures.

In particular, the RAG1/2 DNA recombinase induces somatic deletions and translocations in developing B- and T-lymphocytes, which sometimes dysregulates tumor suppressor and oncogenes, leading to malignant cell transformation and lymphoid cancer. Consistent with the causal relationship between RAG1/2 activity and cancer progression, deficiency of RAG1/2 prevents leukemia development in mouse models. Importantly, even non-lymphoid acute myeloid leukemias (AML) that affect children and young adults can also exhibit complex genomic rearrangements, a subset of which is associated with the expression of RAG1/2 and resultant clonal somatic T-cell receptor rearrangements (10).

Other early acting genomic processes can potentially enhance the mutation rate in developing cells. In particular, APOBEC-family deaminases cause distinct somatic mutations, especially AID, which is activated in developing B-cells. For example, ectopic activation of AID is sufficient to induce mutagenic uracil mismatches, similar to the kataegis mutational signatures observed in distinct human lymphomas (11). In fact, kataegis-associated clustered mutation hotspots in lymphoid cancers contain predominantly AID mutational signatures. While kataegis and RAG1/2-mediated rearrangements have been observed in lymphoid malignancies in both children and adults, their developmental induction explains the peaking incidence of lymphoid leukemias and lymphomas in young children and adults that rapidly decreases with age.

RAG1/2 and AID are restricted in expression to developing lymphocytes and consequently contribute to developmental mutagenesis predominantly in blood cancers. Remarkably, many solid tumors of children and young adults, including medulloblastomas, neuroblastomas, ependymomas, Ewing sarcomas, and rhabdoid tumors, express PGBD5, which is enzymatically related to RAG1/2. Both RAG1/2 and PGBD5 are domesticated DNA transposases that apparently use three aspartic or glutamic acids to mediate somatic sequence-specific DNA rearrangements (12). RAG1/2 expression is restricted to developing lymphocytes, and PGBD5 expression is restricted to neurons and related progenitor cells.

In rhabdoid tumors, which are thought to be derived from developing neuroectodermal progenitor cells, PGBD5 promotes somatic sequence-specific genomic rearrangements, some of which cause inactivating mutations of tumor suppressor genes (13). One mutagenic target is *SMARCB1*, which is the fundamental event in rhabdoid tumor initiation. The expression of PGBD5 in many solid tumors affecting children and young adults may reflect its tendency to induce somatic DNA rearrangements, some of which can be oncogenic, offering potential targets for improved therapy (14). PGBD5 and RAG1/2 have essential functions during normal development, raising the possibility that other domesticated transposases in humans may have both important physiological functions and associated pathogenic consequences. Their functions are currently undefined, but have long been hypothesized (15–17). Their developmental functions can explain the early-onset of RAG1/2 and PGBD5-expressing cancers, but can also promote mutations in older individuals, simply at reduced relative incidence.

In our hypothesis, mutations induced by developmental mutators such as RAG1/2 and PGBD5 that happen early in life lead to specific forms of somatic mosaicism and the risk of early onset cancers. In contrast, mutations induced later in life will be confined to a smaller subset of cells and thus will associate with later onset. This hypothesis emphasizes that developmental mutators may be a primary mechanistic cause of widespread somatic mosaicism, associated with specific sequence and structural mutagenic patterns that arise from their genomic remodeling processes.

The developmental mutator hypothesis predicts that additional mutators will be found that drive the progression of cancers not associated with RAG1/2, AID, and PGBD5 or with inherited mutations, environmental exposures or oncogenic pathogens. Potential examples include infant fibrosarcomas, Wilms tumors, retinoblastomas, osteosarcomas, germ cell tumors, astrocytomas, and other glial tumors and sarcomas that affect children and young adults.

Developmental mutators may include nucleases and enzymes with DNA remodeling activities. Alternatively, they may involve cellular processes that regulate DNA repair and normal mitotic control, such as dysregulation of topoisomerase activity during gene expression or nuclear envelope assembly during cell division, respectively.

This model predicts that particular developmental mutators cause distinct mutational patterns that are characteristic of the associated tumors. Indeed, recent studies identified distinct mutational signatures in specific childhood cancers, for example, the so-called signatures 16 and 18 in pilocytic astrocytomas and rhabdomyosarcomas, respectively (9). Current methods for mutational detection and deconvolution have limited sensitivity, and improved methods will be needed to accurately define oncogenic mutators and test this hypothesis (18). Other developmental processes may also initiate childhood cancers, such as epigenetic modifications that aberrantly reactivate endogenous retroviruses and transposons. High-resolution temporal genomic analysis of tumors compared with corresponding normal tissues should provide clues about the underlying developmental mutators that dominate each kind of early onset cancer.

In summary, one can think of a developmental mutation as intermediate between a germline mutation carried by all cells and a post-development somatic mutation confined to a few cells (5). We expect that, among childhood and early onset adult cancers not associated with germline mutations or mutagen exposure, many cases arise from developmental mutations and somatic mosaicism. Indeed, the interaction among the activity of developmental mutators, environmental exposures, and inherited predisposition also explains the variation in cancer risk among different tissues (19, 20).

Prior studies have related some of the developmental processes we have discussed to cancer. However, the diverse processes of developmental mutators have not previously been unified into a general explanation for non-inherited early onset cancers with predictions about molecular mechanisms, mutational signatures, and somatic mosaicism. The mechanistic insights provided by the developmental mutator model suggest new approaches for the early detection, prevention, and treatment of cancer.

## Author Contributions

AK and SF conceived and wrote this manuscript.

## Conflict of Interest

The authors declare that the research was conducted in the absence of any commercial or financial relationships that could be construed as a potential conflict of interest.
